# Ten Cases Where Pus Was Found in Abdomen*The first five cases of this list have been included in a report of “Recent Abdominal Work,” sent out some three months since. The last five have been done quite recently, some of them within the last few weeks.Some remarks at the close of this article are also taken from that report.

**Published:** 1891-09

**Authors:** T. J. Crofford

**Affiliations:** Memphis, Tenn.


DANIEL'S

### TEXAS MEDICAL JOURNAL.

A Representative Organ of the Medical Profession, and an Exponent of Rational Medicine; devoted to the Organization, Advancement and Elevation of the Profession in Texas.

PUBLISHED MONTHLY.—SUBSCRIPTION $2.00 A YEAR.

Vol. VII. AUSTIN, SEPTEMBER, 1891. No. 3.

### Original Articles.

CONTRIBUTED EXCLUSIVELY TO THIS JOURNAL.

*The Articles in this Department are accepted and published with the understanding that we are not responsible for, nor do we indorse the views and opinions of the writers by so doing.*

For Daniel's Texas Medical Journal.

### HOW I HAVE DEALT WITH MY LAST TEN CASES IN WHICH PUS WAS FOUND IN THE ABDOMEN.*

BY T. J. CROFFORD, M. D., OF MEMPHIS, TENN.

IN making this report I have eliminated all cases of abdominal surgery for other than suppuration. I have not included any case in which it was possible to reach the purulent accumulation through the vaginal summit, but restricted it to those cases encountered within the last twelve months which required abdominal section for the evacuation of a purulent accumulation within the abdominal cavity. Representing the very worst features of abdominal, the worst variety of surgery, some of them had grown desperate, and the operation was a *dernier ressort.*

CASE I. Mrs. G., a multipara, aged thirty-five, was delivered at term; in two weeks or earlier fever set in which did not yield to quinine. Two weeks later I saw the case and found a large but circumscribed swelling on the left side, too high to be felt at the vaginal vault. Diagnosed suppuration. A few days later an incision was made, a pint or more of pus was evacuated, and a drainage tube was put in. Although reduced to an extreme she entirely recovered, and is in all respects well.

CASE II. I first saw Mrs. C., multipara, aged 27 years, on the 24th of last July. One month previously she had been delivered of a seven months dead child. Three days after labor fever set in which had continued up to the time I saw her. Associated with this fever was a circumscribed swelling in the left side, accompanied by great pain. She was weak, and we prepared for an operation that afternoon. In the meantime she was carried to St. Joseph's Hospital, and the operation was done there.

We cautiously opened the abdomen over the swelling into the pus cavity, took advantage of adhesion, evacuated, and put in a drainage tube. She uninterruptedly got well.

CASE III. Mrs. P., a primipara, aged twenty-four, had been a sufferer from pelvic inflamation and its results for several months, dating from the birth of her first and only child. This inflammation had already resulted in suppuration, and the surgeon in attendance had made openings through the vaginal vault and abdominal parieties into the sac of pus which at different times had pointed at these sites. There was also an opening into the bladder from the pus cavity from perforation by the pus. There had also been an opening made by the surgeon into the base of the bladder through the anterior vaginal wall.

At this stage, on November 13th, 1889, I, at the instigation of her physician, took charge of her case. Her pulse was 133, her temperature was 103 degrees; there was delirium from uric poisoning, incident to absorption of the urine in the abdomen.

It was evident that the first thing to be done was to dilate the opening into the sac and draw a tube through the abdominal incision and out through the opening in the vaginal vault. We were enabled to irrigate the abdomen, or that portion taken up by the sac, and in a short while clear up the delirium, and materially improve the condition of the patient. After a treatment in this way for some weeks the opening from the bladder into the abdomen was healed, and all the urine dribbled away through the hole in the base of the bladder. Before a great while this patient was sitting up, and in the course of a few months was

---

* The first five cases of this list have been included in a report of "Recent Abdominal Work," sent out some three months since. The last five have been done quite recently, some of them within the last few weeks.

Some remarks at the close of this article are also taken from that report.

going around, though far from being cured. The pains in the abdomen continued in their accustomed severity. An examination by the bi-manual method showed that there were adhesions, a result of the inflammation through which she had passed. An exploratory incision was offered her, with the idea that if compatible with a reasonable safety, of removing the diseased appendages which had likely produced her trouble and were still offending her. She accepted the proposition. On May 23rd, we opened the abdomen, but found intestines, uterus and appendages so matted together that we did not think it very likely that she would recover if this mass were broken up.

The abdomen was washed out and closed. She was up again in three weeks; has since materially improved; visited her relatives in Canada, and is now attending to her househeld duties.

This case illustrates the value of palliative expectent treatment, and the folly of abandoning treatment because you are foiled in your effort to accomplish what seems to be the proper thing. *Lawson Tate thinks an exploratory operation frequently results in good that can not be explained. Should she suffer too greatly, we would advise the unraveling of this mass of intestines and removing the appendages, although the risk would be great.

CASE IV. Mrs. F., aged 24, came to me September 28, last. She was married one year ago. Her abdomen was quite large. The history was that one year ago an enlargement was presented rather to the left of the median line, and has grown rapidly. The diagnosis was ovarian tumor.

The pulse was high, indicating that suppuration was going on. She was prepared for an operation, and on October 1st, the usual incision was made. The tumor was adherent to the whole front of the abdomen. This was forcibly separated by the hand, the trocar plunged in, and the fluid drawn off. This left the solid portion, which was too large to be removed. The incision was extended to the ensiform cartilage, and the solid portion of the tumor, which weighed eighteen pounds after being separated from its attachments, was lifted out, making with the fluid portion forty pounds in weight. There was quite a lot of pus in the bottom of the sac. Notwithstanding the pulse was more than 100 before operation, 120 for several days thereafter, she made an uninterrupted recovery. I was permitted to examine this case not long since, and am gratified to report her in all respects well.

* Prof. Hadra has expressed the same opinion.—ED.

CASE V. Mrs. S., aged 23, a Jewess, of small stature, has been married three years—no child. Upon examination we diagnosed an ovarian tumor of small size, which was wedged in between uterus and rectum. Each morning at the time of evacuation of the bowels, and for several hours afterward, there was excruciating pain. She was very much reduced in flesh and was decidedly anæmic. The pulse was also high—above 90. Under the influence of local treatment, tonics and massage, we in six weeks had her much improved for the operation of removal of this tumor. On the 9th of this month we opened the abdomen and found the tumor somewhat larger than we anticipated. Upon attempting its removal after loosening its adhesions, to our dismay it ruptured, liberating a pint or two of pus into the abdominal cavity among the intestines. Realizing the great danger from this unfortunate accident, which was not preventable owing to the thinness and rottenness of the walls of the tumor, we removed this pus as rapidly as possible, by means of sponges, and tied off the pedicle. We hastened to make the abdomen clean by repeated irrigations. We had but few patients to progress better than this one has done. She returned to her home on December 7th last in good condition, and subsequent information is to the effect that she is perfectly cured.

CASE VI. Mrs. C. was brought to me on the 7th of last December, suffering from a nodular abdominal tumor the size of a cocoanut or larger. The symptoms and history pointed to a tubercular peritonitis. An exploratory incision was made, a pocket of pus evacuated. The tumor embraced the peritoneum and was likely omental in its origin and could not be removed. She remained under our treatment for two months. During this time there were three or four collections of pus evacuated and the cavity packed with gauze. They healed. She then went to her home. She is somewhat better, but she is still reduced in strength and flesh. An occasional abscess forms which her physician evacuates. It is probable she may eventually recover.

CASE VII. Mrs. M. was on January 5th referred to me by her physician, who, ten days before, had aspirated a large suppurating ovarian cyst. The cyst had again refilled and was greatly disturbing and depressing her. An opening was made below the umbilicus. Owing to almost universal adhesions the cyst wall was stitched to the abdominal, thoroughly washed out and a drainage tube put in. The cavity, which was washed out twice daily, held about one-half gallon. This diminished beautifully from day to day until it (at the expiration of ten days) held only a few ounces. She now was seized with a morbid desire to go home, and all argument for her to remain was worthless. She went home. She returned a few days ago all healed with the exception of a sinus extending just under the skin to the depth of two inches. I advised her to remain two weeks, allowing us to split up the sinus and pack with gauze which would almost certainly cure her. She would not consent to remain from her home more than one week, so we could not agree. She has gained twenty pounds, is able to attend to her household duties, boss the old man and the farm, and is in all respects well, save the sinus just alluded to.

This sinus may heal in the course of a few months without anything being done beyond a cleaning daily.

CASE VIII. Mrs. S., aged thirty-six, was delivered of twins eight months prior to her admission into the Sanitarium, which was March 13. Four days after delivery she had a chill followed by high fevers, rigors, sweating, etc., which told that the pelvic inflammation was approaching suppuration.

This was followed by a discharge of pus through the fallopian tube and womb. When she entered the Sanitarium she was brought on a litter, was exhausted by the high temperature and suppuration which she had been experiencing for near two months, her temperature was still registering high in the afternoon--104 to 105 deg. For several days the interior of the uterus was cleansed by irrigation but the real seat of the suppuration not being reached, the temperature kept up, and her husband who was quite an intelligent and progressive physician, readily agreed to a laparotomy. The abdomen was opened, not in the median line but to the left, above the supposed adhesion, in order that we might determine whether or not they were sufficient to allow of making an opening into the suppurating mass.

We found the adhesions insufficient. The incision was extended downward, the adhesions were broken up in this location, preparatory to moving that whole mass, but other adhesions were found to the region of the pubes, to the womb and the bladder, and to the intestines, so formidable that I was of the opinion that the hemorrhage would be sufficient to kill her in her weakened and anæmic condition. It was also clear to me that the mass was not draining into the womb, because it had gravitated too low, while the woman was on her back.

It not being safe to remove the whole mass, the next best and safest thing to be done was to so place this mass that its suppurating interior might drain through the fallopian tube into the womb.

We accordingly stitched it into the lower portion of the abdominal incision, securing its firm attachment with silk to the parietal peritoneum. Then, after closing the incision above, putting in a glass drainage tube into the peritoneal cavity and packing with gauze down to the mass, we felt that if the case was not cured, there was but little danger from the operation, and in the event it should ever give trouble again, it would be so adherent to the abdominal wall as to give the greater chance of pus coming to the surface. The packing was changed every twenty-four or forty-eight hours, the stitches were removed on the eighth day.

Two weeks ago she returned to her home. Not having had a temperature of more than 100°, and this for only short intervals on the afternoons of the first few days after the operation. Her husband writes me, she is regaining her strength and flesh, and is progressing satisfactorily.

CASE IX. Mrs. L., aged forty, presented herself on the 23d of last month with a large tumor. The abdomen was opened on the next day. It was found to be a multicular ovarian cyst, filled with colloid contents. Some of these cysts had undergone suppuration. These walls containing pus were so thin that when the adhesions were separated, the pus flooded the abdominal cavity. The abdomen was washed for twenty or thirty miuutes with water, a drainage-tube put in and stitched up. In spite of an attack of lagrippe, which brought on bronchitis and pneumonia, she returned to her home last Sunday cured.

CASE X. Mrs. H., aged thirty-two. On the 15th of this month I went to Durant, Miss., to see in consultation a lady who had been quite ill for more than three weeks. Her disease was general peritonitis. The suffering was so great that one-half grain of morphia had to be administered every two or three hours. There were bands of adhesion around the rectum at the sigmoid flexure. No movement of the bowels had taken place for two weeks, not even gas having escaped in this length of time. The drum-like distension was so great that respiration was interfered with to no inconsiderable degree. The pulse, temperature and pallor indicated that unless something be done the end was not far distant.

I proposed a laparatomy and drainage of the peritoneal cavity.

The proposition was accepted. An opening was made wid-way between the umbilicus and pubes, large enough to introduce two fingers, break up the band contracting the gut and guide a Sims' hard rubber tube down in behind the fundus into Douglas' cul-de-sac. This tube was flushed at intervals of a few hours with warm water. In a few hours later gas escaped through the rectum. The intervals grew longer between the doses of morphine; by the next day she was in a condition to stand an effort at removal of the fecal impaction. In one or two more days this was gotten rid of. With the exception of an inflammation of the parotid glands, due to the septic infection which had already taken place, her recovery has been steady.

Her physician writes me she is now out of danger, and will soon be able to sit up.

There are three points connected with the case—1st, the breaking up of the adhesions; 2d, the draining of the peritoneal cavity as any other cavity; 3rd, the influence of the atmospheric pressure directly upon the bowels in overcoming or counteracting the gas within the bowels. The latter point has, so far as I have heard, never been made.

REMARKS.

The fifth case was one in which there was great danger of rupture, which might have occurred at any moment. The cyst wall could be torn like wet paper, and might have given way on slight exertion, such as getting in or out of her carriage. It is impossible to estimate or appreciate such dangers as this lady was daily exposed to.

In this report I have sought not to burden the society with methods, technique and details. This can be brought out in the discussion, as the members may be disposed.

The greatest conservatism compatible with thoroughness has been observed.

In cases 1, 2, 3 and 6, simply the pus was evacuated. It would have been far from conservatism to have attempted a removal of the sac in these cases, whilst in cases 4, 5 and 9 it would have been quite as unsurgical and dangerous to let it remain, although there were some adhesions.

In cases 1, 2, 3, 6, 7, 8 and 10, neither ovary was removed.

In cases 4, 5 and 9, only one of these organs was sacrificed.

Each one has a healthy ovary left, and some of them two. I see no reason why they may not in the future bear children.

The necessity for the removal of the sound ovary is not required in ovariotomy, as some operators claim it is (though I doubt it) in oophorectomy.

In these days of brilliant abdominal snrgery, the operator is too frequently carried beyond the true conservative point, and many a life has been sacrificed in removing, or attempting to remove a suppurating cyst or sac, when if it had only been properly drained the patient could have been saved, and the cure quite as satisfactory. I would say to the young operator, go slow. But upon the other hand, there is nothing like thoroughness of removal of every vestige of the sac, when the peritoneal cavity cannot be shut off from the cavity of suppuration. Here radicalism is true conservatism. I should like in this connection to call attention to the use of sterilized absorbent gauze as a valuable addition to the drainage tube in keeping septic material from the peritoneum. In these operations common sense has predominated. The greatest simplicity in methods has been preserved; boiled water alone has been used for irrigation inside the abdomen.

A long needle invented by myself, for putting in the stitches through both rides at once, has been used. I am satisfied that in closing a long incision, 10, 20 or 30 minutes can be saved. Time at this junction is very valuable in obviating, as far as possible, the oncoming shock. The needle is pushed down through the side next the operator; then, with the aid of the assistant, up through the opposite side. A suture is placed in the slot; it is then quickly withdrawn, bringing with it the suture. This needle differs from all others in having a length and curve adapted to putting in stitches through both sides of the abdominal wall at one stroke or movement. All sutures, one after the other, are introduced in the manner described.

With the exception of the spray, the most thorough antisepsis outside, and asepsis inside the abdomen has been observed.

It is perhaps worth stating that antisepsis, with me, is only valuable as it conduces to asepsis. I believe in the use of the spray before the operation, not that the carbolic acid in it is of value in killing germs, not that it is so important to kill germs; not this at all—but I do believe by increasing the humidity of the atmosphere, it will cause the organic matter floating in the room to settle down, and there is consequently less likelihood of its being deposited in the open wound.

It is gratifying to me to relate the fact that I have never had a case of peritonitis to follow an abdominal operation, nor has a ventral hernia ever ensued.

